# Sharp force trauma analysis without animal bones: A proposal for sustainable and ethical bone proxies

**DOI:** 10.1111/1556-4029.70402

**Published:** 2026-07-02

**Authors:** Beth Lawrence, Giada Sciâdi Steiger, Matteo Borrini

**Affiliations:** ^1^ School of Biological and Environmental Sciences Liverpool John Moores University Liverpool UK

**Keywords:** animal bone proxies, cut‐mark analysis, ethical considerations, ethical research practices, forensic anthropology, serrated knife, sharp force trauma

## Abstract

Sharp force trauma has been the leading cause of homicide in England and Wales for more than a decade. Experimental research on sharp force trauma frequently relies on animal bone as a substitute for human remains, yet their ethical acceptability and comparability to human bone remain contested. This study aims to identify non‐animal proxy materials capable of replicating the morphological characteristics of cut marks typically observed in porcine bones. A total of 250 cuts were made on pig ribs and on several proxy materials (paraffin, restoration compound, plaster, and beeswax) using a serrated knife. Six morphological traits were then recorded following the analytical framework proposed by Steiger and Borrini (*J Forensic Sci*, 2024;69:1972). Chi‐squared tests of independence and mixed‐model analyses demonstrated that different proxy materials vary in their suitability for specific traits, and that among all tested materials, beeswax most closely replicated porcine bone in the context of cut marks produced with a serrated blade. Considering the ethical implications of using animal bone, this research proposes sustainable, cruelty‐free alternatives for experimental and educational purposes. Their adoption reflects contemporary ethical awareness regarding animal use in science and promotes ecological and economic sustainability through reusability.

**Highlights**

- Cruelty‐free sustainable materials were tested as substitutes for animal bone in cut mark analysis.
- Non‐bone proxies provide accessible, reusable, and ethically sourced options for forensic tests.
- These materials support reproducible research and practical forensic training.

## 1 INTRODUCTION

Sharp force trauma represents the most common form of homicide in England and Wales and has remained the leading cause for over a decade, fluctuating between 36% and 42%, with 41% of homicides in 2022 involving knives, according to the Office for National Statistics [1]. Globally, homicides caused by sharp objects accounted for 22% of deaths in 2017, representing over one‐third of homicides in Asia [1]. Given these statistics, the analysis and interpretation of sharp force trauma can provide critical contextual evidence in forensic investigations.

Extensive research has been conducted on sharp force trauma on bone, exploring how cut marks are affected by thermal alteration and taphonomic processes, and describing dismemberment patterns, tool marks, and cut mark morphology [2, 3, 4, 5, 6, 7, 8, 9, 10, 11, 12, 13, 14, 15, 16, 17]. Previous experiments have focused primarily on generalized tool‐mark features, known as class characteristics [2, 17, 18, 19], with primary class characteristics including the type of impression, shape, and dimensions of the cut, whereas secondary characteristics encompass bone fragmentation and fracturing [7, 8, 10, 13, 20, 21]. These traits provide investigators with general information regarding the weapon class but do not offer conclusive evidence of a specific implement. Other authors have examined weapon‐specific characteristics, including blade damage resulting from use and the corresponding variations in cut mark patterns observed on bone [16, 22, 23, 24, 25], while additional studies have analyzed injuries documented through radiography or postmortem examination [26, 27].

The literature demonstrates that animal proxies, particularly porcine (*Sus scrofa*) ribs, have been widely employed in experimental research to better understand the dynamics of sharp force trauma and the marks left on bone due to their anatomical and physiological similarities, as well as their accessibility [4, 7, 8]. However, while numerous studies have reported successful outcomes using animal proxies in various forensic applications [28, 29, 30, 31, 32], questions remain regarding their accuracy and ethical implications [33, 34, 35]. In this context, ethical implications refer specifically to concerns regarding animal welfare, the sustainability of animal‐based practices, and potential conflicts between institutional requirements and individual moral convictions.

The use of animals in forensic research raises significant ethical concerns, particularly given that biological tissue is a finite resource and that its procurement and use may involve pain, distress, or death [33]. Although post‐mortem animal specimens—often by‐products of the food industry—are the ones most frequently employed in forensic studies, their continued reliance nevertheless underscores the need to identify and validate alternative, non‐animal materials capable of providing comparable scientific value [33]. Even where specimens are sourced as by‐products, their use implies the continued consumption of finite biological resources and may contribute to material waste when samples cannot be reused following experimental procedures. By contrast, reusable synthetic or wax‐based proxies offer practical and ethical advantages. They can be repeatedly cast and reshaped to replicate trauma patterns with consistency, enabling their use across multiple experiments and related studies with varying objectives. This characteristic not only reduces overall material consumption but also enhances experimental standardization while lowering long‐term costs.

The use of animal proxies may also generate ethical concerns among communities that emphasize compassion and responsible treatment of animals, particularly when limited information is available regarding how the specimens are sourced and whether their use aligns with established animal welfare principles [34]. These concerns are particularly relevant within contemporary forensic anthropology, where, as Borgelt et al. [36] argue, increasing diversity within the discipline necessitates critical examination of rooted pedagogical norms that may function as systemic barriers to inclusion. The compulsory handling of animal bone, while traditionally regarded as possible standard training practice, may generate moral, cultural, or religious conflicts for some individuals, including students who adhere to vegan or vegetarian principles. In light of the authors' emphasis [36] on equity, belonging, and structural reform within forensic anthropology education, the development and validation of cruelty‐free proxies represent not merely logistical accommodation but a meaningful step toward culturally responsive and ethically inclusive pedagogy. Providing equivalent hands‐on alternatives would allow institutions to uphold rigorous training standards while aligning with broader commitments to diversity, equity, and responsible disciplinary practice.

Developing and adopting such alternatives is essential to reduce animal use, minimize ethical and welfare impacts, and improve the sustainability of research practices while still ensuring that robust and reliable scientific outcomes are achieved.

The following section provides an overview of selected non‐animal materials that have been suggested or utilized in previous studies across various contexts, including skeletal reconstruction and medical settings, highlighting their potential suitability for use in trauma analysis research as animal bone substitutes.

### 1.1 Synbone

A widely used synthetic substitute known as Synthetic Bone, or Synbone, was developed to replicate the human skull and macroscopic biomechanical responses. Synbone has been employed extensively in both ballistic and blunt force trauma research, contributing valuable insights to forensic investigations [37, 38, 39, 40, 41] and archaeological studies [42, 43, 44, 45].

### 1.2 Wax

Only a limited number of studies have examined casting wax as a medium for tool‐mark analysis; nevertheless, promising results have been reported using different types (e.g., jewelry, dental, and modeling wax), demonstrating that fine morphological details can be effectively recorded [10, 46]. In a study conducted by Crowder and colleagues [10], experts successfully evaluated knife marks in casting wax made with serrated, non‐serrated, and partially serrated blades; however, they concluded that the impressions made on hardened casting wax were unreliable due to brittleness under pressure [10].

One of the candidate proxies in the present study is the restoration compound, a wax‐based material formulated by Borrini [47] and described in detail by Valoriani and colleagues [48]. The compound, originally developed for reconstructing fragmented skulls, is reversible and reusable, allowing it to be stored for future experiments. It consists of two wax types combined with additional components to produce a stable, protective, and easily moldable surface suitable for analysis [8].

Additionally, paraffin and beeswax are widely used in surgical practice to prevent bleeding, and a mixture of beeswax with a softening agent—known as bone wax—has long been used to seal bone fractures [49]. Soft waxes maintain structural integrity under pressure, making them ideal for recording fine tool‐mark impressions.

### 1.3 Plaster

Plaster of Paris is another commonly used substitute for bone due to its availability, low cost, and ease of handling [50]. Although originally used in bone regeneration as a substitute, it is now primarily applied as a temporary filler or carrier for other grafts due to its rapid resorption rate [51]. In forensic contexts, plaster has proven valuable for impression analysis, particularly because of its rapid setting properties, which make it advantageous for recording evidence at crime scenes. More specifically, it has been used to preserve footwear impressions, tire marks, bite marks, and tool marks, or to capture detailed lesion morphology, providing a durable record of trauma [52, 53, 54].

The present study therefore aims to identify cruelty‐free and sustainable alternatives among beeswax, the restoration compound, paraffin, and plaster for cut mark analysis. Specifically, it seeks to evaluate these materials as potential proxies for human and animal bone in experimental studies of sharp force trauma and tool‐mark identification relevant to forensic and archaeological investigations.

## 2 MATERIALS AND METHODS

Four materials were investigated and compared with animal bone: beeswax, paraffin wax, plaster of Paris, and the restoration compound. Synbone was excluded from this analysis, as its large‐scale use is limited by cost and restricted availability.

Sample one (ribs) consisted of five porcine ribs (*Sus scrofa*) housed at Liverpool John Moores University. These specimens were part of a previous study that established a methodology for assessing the morphological traits of cut marks [55]. Sample two (beeswax) was prepared using 200 g of yellow beeswax pellets, melted on a metallic hotplate, and stirred with a spatula as needed to prevent burning. Once fully liquefied, the wax was carefully poured into molds to produce five semi‐cylindrical casts. The same molds were used for samples three, four, and five.

**FIGURE 1:**
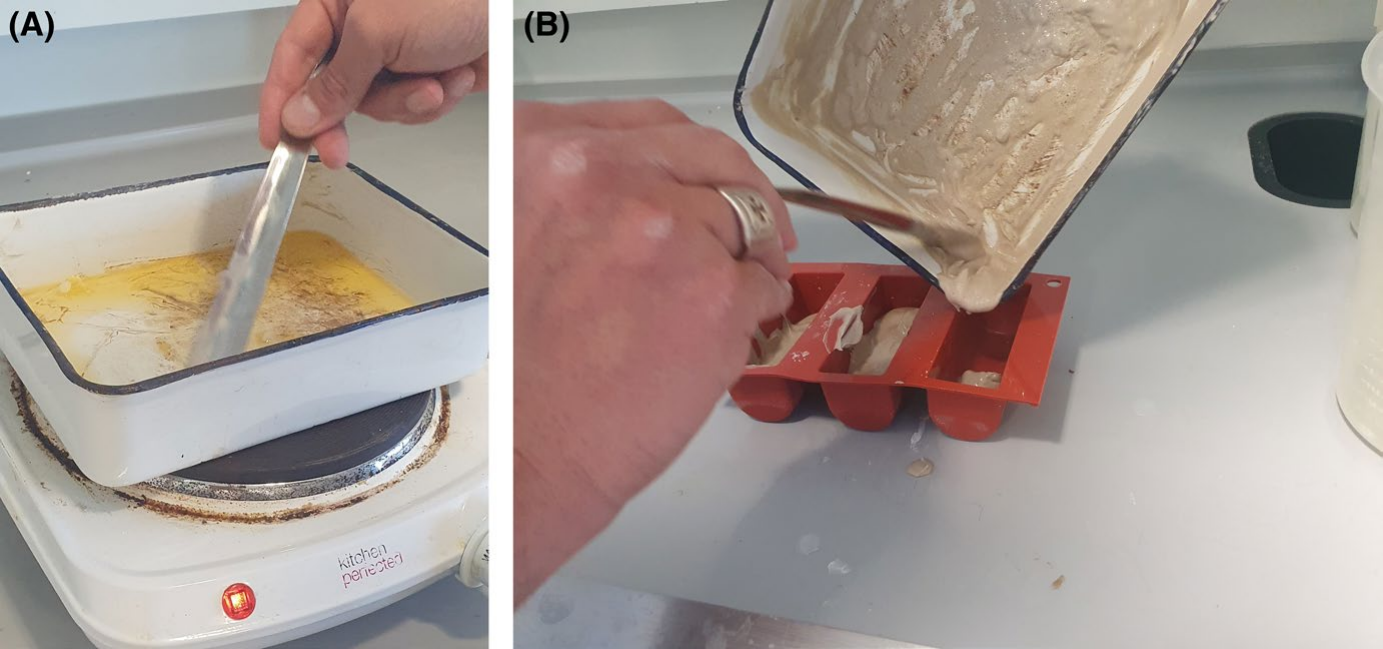
(A) The restoration compound components are mixed in a metal tray and melted using a hot plate. (B) The restoration compound is poured into a bone mold.

Sample three (paraffin wax) was prepared using the same procedure as sample two. For sample four (plaster), 32 mL of water was gradually added to 100 g of plaster and gently mixed to create a smooth paste. The mixture was poured into the molds to produce five casts, gently tapped to release air bubbles, and allowed to solidify. For sample five (the restoration compound), several components were combined in a metallic tray on a hotplate (Figure 1A). First, 20 g of beeswax and 10 g of pine rosin were melted and stirred to prevent solid residues. Once a uniform blend was achieved, 20 g of paraffin (candle wax), 60 g of casting powder, and 60 g of calcium carbonate (limestone flour) were added. After homogenization, the compound was poured into the molds to form five casts, which were left to cool and solidify (Figure 1B).

**FIGURE 2:**
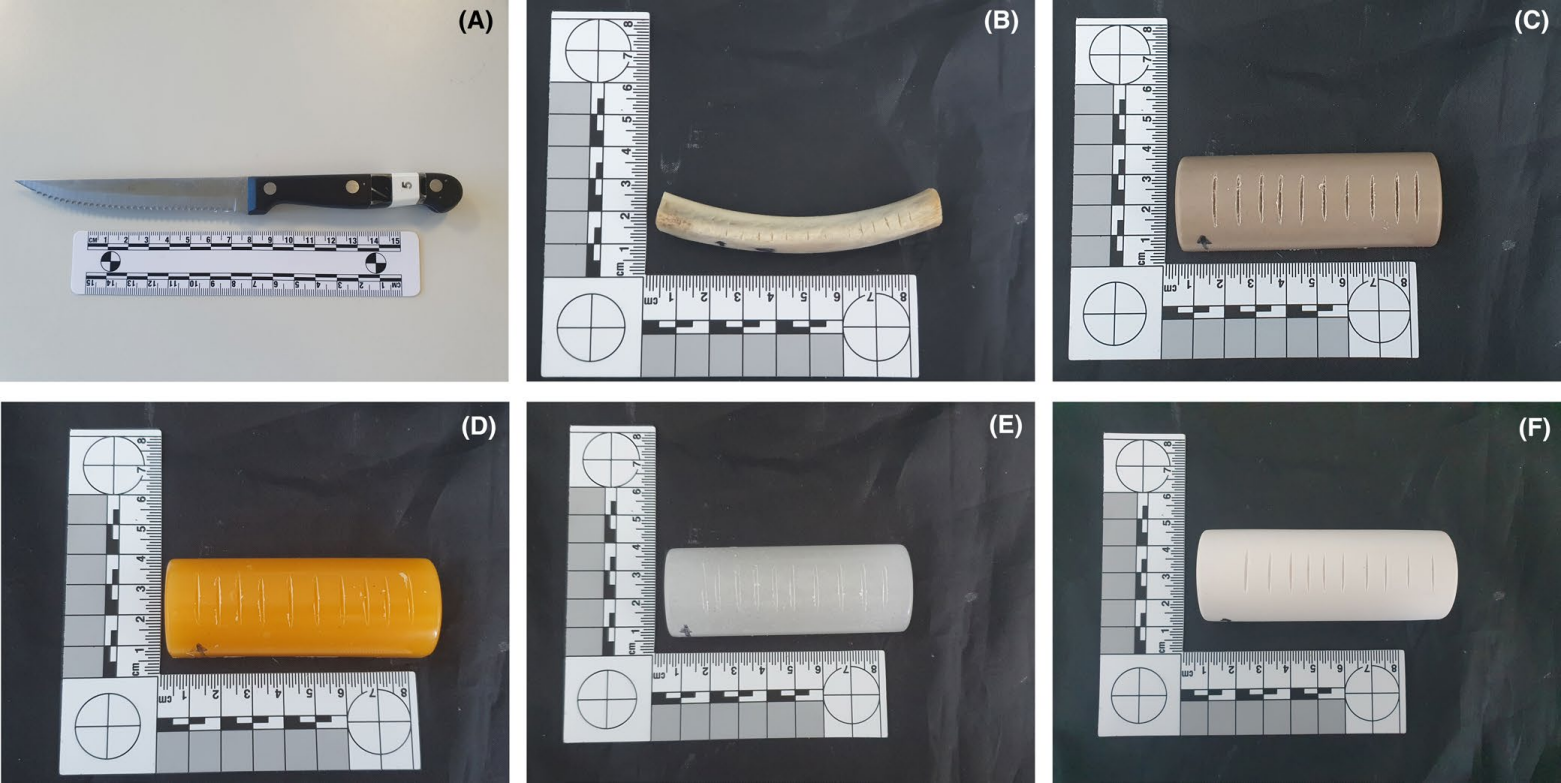
Materials that were used in this study. (A) The serrated knife that was used to create cut marks in each of the samples. (B) One of the porcine rib samples that was taken from Steiger and Borrini's study [55]. (C) Example of the restoration compound cast showing 10 cut marks. (D) Example of the beeswax cast showing 10 cut marks. (E) Example of the paraffin cast showing 10 cut marks. (F) Example of the plaster cast showing 10 cut marks.

To simulate stab wounds to the thoracic cage, 10 cut marks were generated per cast using a serrated knife (Figure 2A) in a single forward–backward action. The serrated knife was selected based on prior research [55], which identified this blade type as producing cut marks most representative of the serrated class and exhibiting the highest degree of morphological clarity. In total, 200 new cuts were generated, producing 250 cut marks. The same external researcher who prepared the samples for the original study [55] performed all cuts to ensure consistency in handling and applied force. Representative examples of each proxy are shown in Figure 2b–f.

Analyses were conducted using a stereomicroscope at 38× magnification. Six morphological traits were recorded and categorized following the terminology proposed by Steiger and Borrini [55]:

- Shape: the general morphology of the cut mark (kerf) was classified as Elliptical (Shape E), D‐shaped (Shape D), or Indeterminate (Shape I) if it did not clearly fit either category.
- Cross‐profile: the cross‐sectional profile shape was recorded as either V‐shaped (V shape) or Y‐shaped (Y shape).
- Rising: this characteristic is identified by looking at the cross‐profile. The trait was recorded if present, as either single (rising single) or bilateral (rising bilateral). If no rise was observed, the trait was recorded as absent (rising absent).
- Shards: the presence or absence of any shards on the cut mark was recorded.
- Feathering: the presence or absence of any feathering on the cut mark was recorded.
- Mounding: this characteristic is identified by looking at the surface of the cut mark. The trait was recorded if present, as either marked (mounding marked) or not marked (mounding not marked). If no mounding was present, this trait was recorded as absent (mounding absent).

**FIGURE 3:**
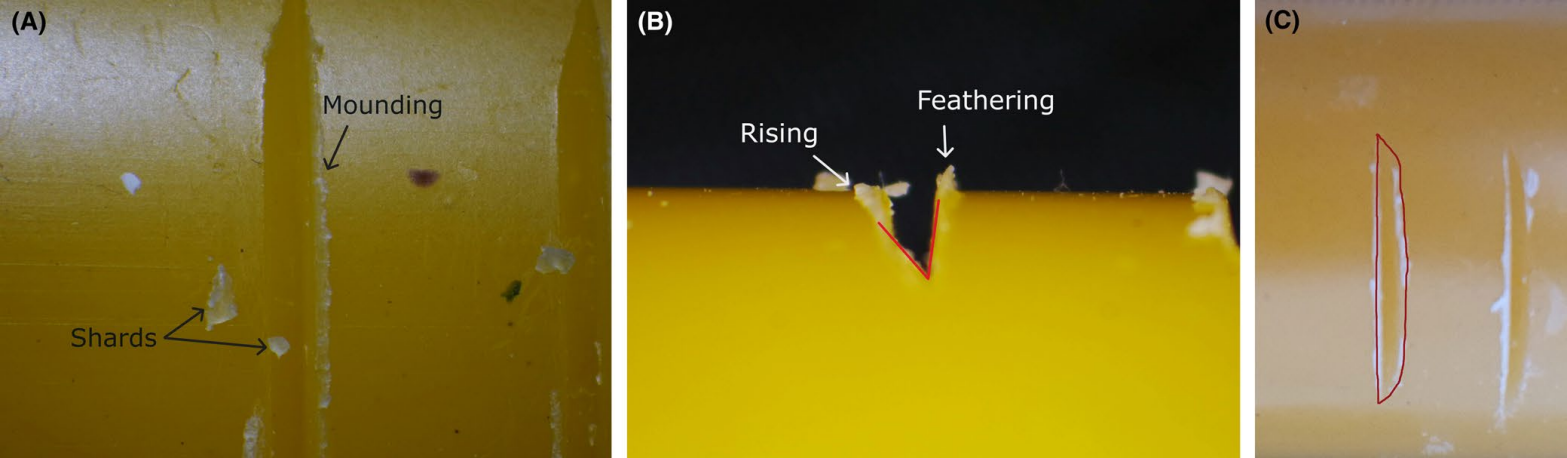
Morphological features that are associated with a serrated knife. (A) Shards present and mounding marked. (B) Rising single and feathering, with the cross‐profile Y shape. (C) Shape D of the cut mark following the kerf margins.

Figure 3 illustrates the categories of traits associated with the serrated knife.

**FIGURE 4:**
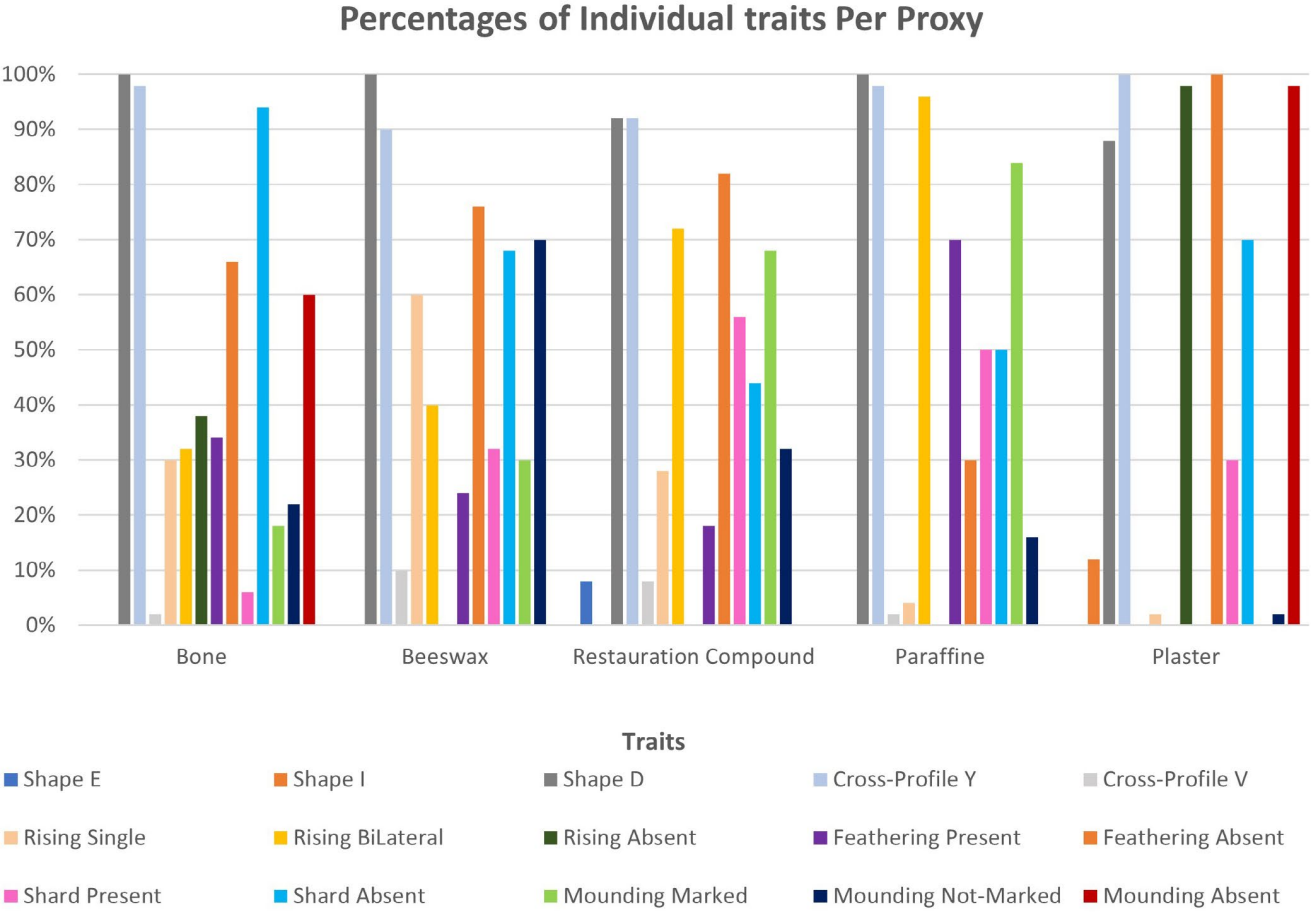
The clustered bar chart shows the morphological characteristics described by Steiger and Borrini [55] and the classification percentages for each proxy.

Data were compiled in Microsoft® Excel® for Microsoft 365 MSO (Version 2307 Build 16.0.16626.20170). The sum of all variables was calculated, and percentage distributions were tabulated to generate the graph presented in Figure 4. Statistical analyses were performed using Jamovi 2.6.44. Each morphological category was analyzed independently to determine what features were similar to the bone reference.

First, Cohen's kappa was calculated to evaluate the intra‐observer error between measurements taken by the same observer 2 weeks apart. Data for each proxy were collected from all five casts at cut marks 1, 5, and 10. Subsequently, initial comparisons between material and feature were conducted using a chi‐squared test. To further investigate where differences were between materials, a linear mixed model was used based on the proportion of a feature per sample, so that cut marks were not treated as independent.

To control for the increased risk of Type I errors associated with multiple comparisons, *p*‐values were adjusted using the Benjamini–Hochberg procedure, which limits the false discovery rate and reduces the likelihood that statistically significant results arise by chance [56].

## 3 RESULTS

The analysis for rater reliability was classed as follows: moderate for shape, feathering, and mounding; fair for shards; almost perfect for rising; and poor for cross‐profile.

Although Cohen's kappa for cross‐profile was poor, the percent agreement was 94.7%. Because the same rater recorded the traits, guessing and rater variability were unlikely; therefore, cross‐profile was retained as a variable.

The analysis of comparisons from the chi‐squared test of independence determined that only cross‐profile is independent of material type (*p >* 0.05). In contrast, all other features demonstrated significant dependence on material.

The linear mixed model was used to closely analyze where differences were between material and feature type, and whether a material was determined to be different from bone. Feature type accounted for most of the observed variability (*p* < 0.001) for shape, cross‐profile, shards, and feathering (Table 1). When all features were analyzed together, none of the materials differed significantly from bone (*p* = 1.000). However, significant feature‐by‐material interactions were observed for all features except cross‐profile, indicating that differences between materials depended on the feature being evaluated. Cross‐profile again appeared consistent across materials, showing no evidence of interaction.

**TABLE 1:** Statistical results of fixed effects omnibus test analyzing the significance of the main effects of feature and material, and if there were differences between material and feature compared to bone.

Feature	Main effect of feature	Main effect of material (proxy)	Feature × material interaction
Shape	*F*(2,60) = 1416.10, *p* < 0.001	*F*(4,60)≈0, *p* = 1.000	*F*(8,60) = 3.54, *p* = 0.002
Cross‐profile	*F*(1,40) = 1019.29, *p* < 0.001	*F*(4,40)≈0, *p* = 1.000	*F*(4,40) = 1.84, *p* = 0.140
Shards	*F*(1,40) = 39.0, *p* < 0.001	*F*(4,40)≈0, *p* = 1.000	*F*(4,40) = 13.0, *p* < 0.001
Mounding	*F*(2,60) = 2.82, *p* = 0.068	*F*(4,60) = 7.08, *p* < 1.000	*F*(8,60) = 30.89, *p* < 0.001
Rising	*F*(2,40) = 0.897, *p* = 0.416	*F*(4,20) = 1.00, *p* = 0.431	*F*(8,40) = 3.717, *p* = 0.002
Feathering	*F*(1,40) = 43.6, *p* < 0.001	*F*(4,40)≈0, *p* = 1.000	*F*(4,40) = 13.6, *p* < 0.001

Parameter estimates show which materials differ from bone for each feature. Only the restoration compound was found to be significantly different from bone for Shape D (*p* = 0.128). Whereas both beeswax and paraffin replicate the same proportions as bone for shape (*p* = 1.000) (Table 2). More variation was seen for the feature mounding (Table 3), with only beeswax being most similar to bone for not marked versus marked (*Δ* = 0.360, *p* = 0.032), although statistically still significant, and plaster, which had very similar proportions to bone (*Δ* = −0.020, *p* = 0.901). For absent versus marked, all materials were determined to be significantly different from bone, with only plaster showing a notably higher proportion (*Δ* = 0.56, *p* = 0.002).

**TABLE 2:** Parameter estimates for Shape D vs. Shape E, and Shape I vs. Shape E against material compared to bone proportions.

Shape	Material	*Δ* proportion vs. bone	*p*‐value
D vs. E	Beeswax	0.000	1.000
D vs. E	Paraffin	0.000	1.000
D vs. E	Restoration compound	−0.160	0.128
D vs. E	Plaster	−0.120	0.181
I vs. E	Beeswax	0.008	1.000
I vs. E	Paraffin	0.000	1.000
I vs. E	Restoration compound	−0.080	0.440
I vs. E	Plaster	0.120	0.181

**TABLE 3:** Parameter estimates for mounding not marked vs. marked and absent vs. marked for materials compared to bone using proportions.

Mounding	Material	*Δ* proportion vs. bone	*p*‐value
Not marked vs. Marked	Beeswax	0.36	0.032
Not marked vs. Marked	Paraffin	−0.720	0.002
Not marked vs. Marked	Restoration compound	−0.400	0.020
Not marked vs. Marked	Plaster	−0.020	0.901
Absent vs. Marked	Beeswax	−0.720	0.002
Absent vs. Marked	Paraffin	−1.260	0.002
Absent vs. Marked	Restoration compound	−1.100	0.002
Absent vs. Marked	Plaster	0.56	0.002

For rising, there were no significant differences when comparing rising single to rising bilateral across materials. However, plaster showed a significant difference for rising single versus absent (*Δ* = 2.500, *p* = 0.040) (Table 4).

**TABLE 4:** Parameter estimates for rising bilateral vs. single and rising absent vs. single for materials compared to bone using proportions.

Rising	Material	*Δ* proportion vs. bone	*p*‐value
Rising bilateral vs. Single	Beeswax	−0.220	0.962
Rising bilateral vs. Single	Restoration compound	0.420	0.962
Rising bilateral vs. Single	Paraffin	0.900	0.962
Rising bilateral vs. Single	Plaster	−0.040	0.962
Rising absent vs. Single	Beeswax	−0.680	0.962
Rising absent vs. Single	Restoration compound	−0.360	0.962
Rising absent vs. Single	Paraffin	−0.120	0.962
Rising absent vs. Single	Plaster	2.500	0.040

For cross‐profile, none of the materials differed significantly from bone (Table 4 and Table 5). For the cross–profile, the largest deviation was seen in beeswax (*Δ* = −0.160, *p* = 0.336), but this was still not statistically significant.

**TABLE 5:** Parameter estimates for cross‐profile Y vs. V and materials compared to bone using proportions.

Cross‐profile (Y vs. V)	Material	*Δ* proportion vs. bone	*p*‐value
Y vs. V	Beeswax	−0.160	0.336
Y vs. V	Restoration compound	−0.120	0.384
Y vs. V	Paraffin	0.000	1.000
Y vs. V	Plaster	0.040	0.880

The presence versus absence of feathering (Table 6) was similar to bone for both beeswax (*Δ* = 0.200, *p* = 0.321) and the restoration compound (*Δ* = 0.320, *p* = 0.155), with only slightly higher proportions.

**TABLE 6:** Parameter estimates for feathering presence vs. absence of materials compared to bone using proportions.

Feathering	Material	*Δ* proportion vs. bone	*p*‐value
Absent vs. Present	Beeswax	0.200	0.321
Absent vs. Present	Restoration compound	0.320	0.155
Absent vs. Present	Paraffin	−0.720	0.002
Absent vs. Present	Plaster	0.680	0.002

For shards, all materials had significantly fewer shards than bone, with plaster deviating the least (*Δ* = −0.480, *p* = 0.003) and the restoration compound deviating the most (*Δ* = −1.000, *p* = 0.002) (Table 7).

**TABLE 7:** Parameter estimates for shards presence vs. absence of materials compared to bone using proportions.

Shards	Material	*Δ* proportion vs. bone	*p*‐value
Absent vs. Present	Beeswax	−0.520	0.003
Absent vs. Present	Restoration compound	−1.000	0.002
Absent vs. Present	Paraffin	−0.880	0.002
Absent vs. Present	Plaster	−0.480	0.003

## 4 DISCUSSION

The study aimed to identify cruelty‐free bone proxies for cut mark analysis by comparing their ability to show standardized traits with animal bones. The results showed that, although several traits differed significantly among the four proxies and the reference porcine bone, certain similarities were observed. Specifically, plaster differed from bone for feathering, shards, and one mounding category and one rising category; beeswax differed for shards and both mounding categories; paraffin differed for both mounding categories, feathering, and shards; the restoration compound differed for one shape category, both mounding categories, and shards. Beeswax replicated 6 of 9 trait categories, while the remaining proxies replicated 5 of 9, indicating that beeswax achieved the closest morphological correspondence to animal bone.

Analysis of individual trait percentages revealed high variability in rising and mounding across beeswax, the restoration compound, and paraffin. However, this degree of variation was comparable to that observed in porcine bone (e.g., variations in the distribution of rising: 30% single, 32% bilateral, 38% absent), indicating that such variability represents an intrinsic characteristic of the cut marks themselves. For the trait mounding, porcine bone showed 18% marked, 22% not marked, and 60% absent, demonstrating no dominant expression of the trait; comparable variation was evident in beeswax, the restoration compound, and paraffin.

Shards were more frequently observed in all proxies than in porcine bone, where they were absent in over 90% of cut marks (Figure 4). Previous studies suggest that shards can occur along the straight bevel of cuts from serrated knives [4, 9], supporting these observations. The restoration compound displayed a higher frequency of shards, likely due to the condition of the samples, which were part of earlier research [55]. Over time, small fragments may have been lost during storage, potentially reducing shard frequency in the bone samples.

Regarding shape, previous research has associated the D shape with serrated blades [4, 7, 9, 13]. In this study, beeswax and paraffin accurately replicated bone in this category, as the relative frequency of this trait (100%) was the same for both media. The restoration compound also showed high similarity (>90% of cut marks showed this category), whereas plaster differed significantly from porcine bone, suggesting it is unsuitable for replicating the shape characteristic of serrated knife cut marks. Results for feathering showed no significant differences between beeswax, the restoration compound, and bone, and the strongest overall correspondence was observed in the cross‐profile analysis, where all proxies demonstrated a high degree of similarity to bone, indicating their suitability for analytical and experimental use.

### 4.1 Implications

Non‐bone materials can effectively reproduce many of the morphological characteristics associated with serrated knives. This is particularly evident in beeswax, where 6 of the 9 trait categories exhibited no statistically significant differences from porcine bone. While certain materials may be more suitable for examining specific traits (e.g., plaster for mounding), a comprehensive, integrative approach encompassing the full range of features that define a cut mark is preferable. The combined assessment of multiple characteristics enhances the precision and reliability of weapon identification [27, 55]; consequently, proxies that most closely replicate the majority of diagnostic traits should be selected.

The capacity to reproduce injury patterns using cruelty‐free and sustainable alternatives to human and animal bone is promising and would enable forensic scientists and anthropologists to simulate trauma with high fidelity while minimizing ethical concerns. A prior case study demonstrated that wax impressions of cut marks obtained at a crime scene could be successfully matched to specific swords [57]; therefore, the material that yielded the most consistent results in the present study may be applicable in similar investigative settings. This technique also holds relevance in historical research, where behavioral reconstructions—such as analyses of butchery practices—can be conducted through the examination of tool marks [2, 32, 45].

Traditionally, experimental replication of trauma has relied on fresh bone specimens that are sectioned immediately preceding analysis [58, 59]; however, the age of the slaughtered animal is a critical factor to consider. Prior research [60] demonstrated that although porcine ribs represent a closer analogue to human bone than caprine ribs, the developmental stage of the animal substantially affects comparability. Because pigs in the meat industry are generally slaughtered at a young age, their osseous structure remains incompletely developed. Ribs from older breeding pigs, characterized by a higher proportion of lamellar bone, more closely approximate adult human rib tissue and thus improve analytical accuracy [61]. Nonetheless, obtaining ribs from older pigs is often logistically challenging due to limited availability. The use of proxies such as beeswax, as proposed in this study, offers a practical solution to these logistical and sourcing constraints.

Beyond their methodological advantages, the adoption of such proxies reflects an evolving ethical framework within forensic and anthropological research. Although beeswax‐based materials cannot be classified as fully vegan, they are cruelty‐free, as their use does not involve the harming of animals. This shift aligns with broader scientific trends emphasizing the reduction or replacement of animal‐derived materials in experimental and educational contexts [33]. Moreover, the avoidance of animal bone could mitigate religious and cultural concerns that may arise from the use of animal remains. Consequently, the use of non‐animal proxies is well‐suited for application in research and investigative environments that prioritize ethical integrity and cultural sensitivity.

In addition, the use of cruelty‐free proxies offers substantial value in forensic and anthropological training. Traditional instruction in sharp force trauma analysis often relies on limited animal or human bone material, which can pose ethical and logistical challenges. The use of beeswax provides a practical and reproducible alternative for hands‐on teaching, allowing students to observe and document tool mark morphology under controlled and repeatable conditions. Its physical properties also permit realistic simulation of weapon–bone interactions, enhancing experiential learning and improving consistency across training programs. Finally, this material can be remolded and recut, enabling standardized exercises that support skill development while maintaining compliance with institutional ethics policies and making beeswax a sustainable option both environmentally and economically, aligning with the Replacement principle of the 3Rs proposed by Russell and Burch in 1959 [62].

Future research should further assess the applicability of these proxies under a variety of controlled experimental conditions, including machine‐assisted cutting systems. Mechanically regulated methods provide precise control over parameters such as force, velocity, and angle [63, 64, 65, 66], thereby minimizing inconsistencies associated with manual cutting and facilitating the isolation of specific morphological attributes relevant to weapon identification. Although mechanical cutting may not fully replicate real‐world trauma dynamics, observed differences between manual and automated marks underscore the importance of parameter control in subsequent investigations [67]. Additionally, biomechanical testing on proxies subjected to repeated melting and remolding would be valuable in evaluating their durability and long‐term performance.

## 5 CONCLUSION

The present study sought to identify cruelty‐free and sustainable proxies suitable for replicating serrated‐knife–induced cut marks through a comparative analysis of their capacity to reproduce standardized morphological traits observed in animal bone. The findings demonstrate that beeswax was the most reliable among the four materials tested, exhibiting close morphological correspondence with porcine bone across multiple diagnostic features.

The use of non‐bone proxies represents a promising advancement in the methodological and ethical dimensions of experimental forensic research. By reducing dependence on animal‐derived specimens, these materials address long‐standing ethical concerns, reflect contemporary sensitivity toward animal welfare, and accommodate diverse cultural and religious considerations regarding the use of animal remains. Moreover, their reusability enhances ecological and economic sustainability, offering a practical alternative for both research and pedagogical applications.

This study establishes a foundation for further investigation into the reproducibility and diagnostic reliability of cut mark traits generated on non‐bone substrates. Future research should evaluate the performance of these proxies in broader experimental contexts, including other types of knives, various weapon types, mechanical cutting systems, and repeated use cycles, to refine their applicability in forensic, archaeological, and experimental settings.

## CONFLICT OF INTEREST STATEMENT

The authors declare no conflicts of interest.

## Data Availability

The data that support the findings of this study are available from the corresponding author upon reasonable request.
